# Synthesis, crystal structure, Hirshfeld surface analysis, and DFT calculation of 4-(5-(((1-(3,4,5-trimethoxyphenyl)-1*H*-1,2,3-triazol-4-yl)methyl)thio)-4-phenyl-4*H*-1,2,4-triazol-3-yl)pyridine

**DOI:** 10.1016/j.heliyon.2024.e40318

**Published:** 2024-11-12

**Authors:** Mohamed El-Naggar, Kamrul Hasan, Monther A. Khanfar, Fatima-Azzahra Delmani, Ihsan A. Shehadi, Raed Al-Qawasmeh, Hussein M. Elmehdi

**Affiliations:** a Chemistry Department, Faculty of Sciences, Pure and Applied Chemistry Group, University of Sharjah, P. O. Box 27272, Sharjah, United Arab Emirates; b Department of Chemistry, The University of Jordan, Amman, 11942, Jordan; c Department of Science, Faculty of Science, Jerash University, 26150, Jerash, Jordan; d Department of Applied Physics and Astronomy, University of Sharjah, Sharjah, 27272, United Arab Emirates

**Keywords:** 1,2,3-Triazole, Pyridine, X-ray structure, Hirshfeld analysis, And DFT

## Abstract

Triazole is considered as a privileged scaffold in medicinal chemistry by virtue of it is diverse biological activity. several drugs currently in the market possess triazole moiety. In this study click chemistry was performed on the pyridine based 1,2,4-triazole-tethered propargyl moiety to afford 4-(5-(((1-(3,4,5-trimethoxyphenyl)-1*H*-1,2,3-triazol-4-yl)methyl)thio)-4-phenyl-4*H*-1,2,4-triazol-3-yl)pyridine**.** The new compound was fully characterized by ^1^H NMR, ^13^C NMR, HRMS and X-ray diffraction (XRD). XRD data indicated that, the structure shows: triclinic, space group P −1, a = 6.4427(3) A, ° b = 11.4352(4) A, ° c = 15.4510(5) A, ° α = 97.980(2)°, β = 96.043(2)°, γ = 92.772(2)°, V = 1118.75(7) Å 3, Z = 2, T = 152(2) K, μ(MoKα) = 0.094 mm−1, Dcalc = 1.364 g/cm3. Density functional theory (DFT) method along with Hirshfeld analysis of the optimized X-ray structure of the final product were used to confirm the molecular and the electronic structure of the reported compound.

## Graphical abstract

**Figure:**
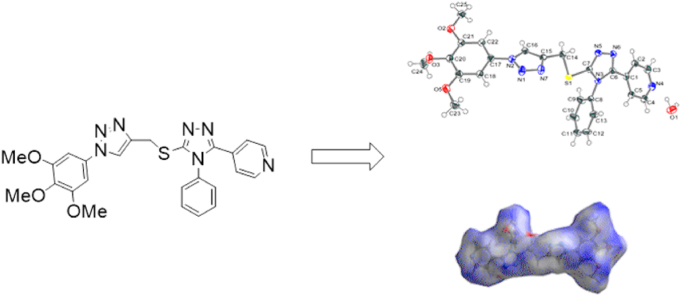

## 1 Introduction

Heterocyclic organic chemistry is one of the most important and well-studied branches of medicinal chemistry. Nitrogen based heterocyclic compounds encompass fascinating pharmacological and biological studies such as antibacterial, antifungal, antitubercular, antioxidant and antitumor agents [1,2]. Among the nitrogen heterocyclic compounds are triazoles, family of five-membered heterocyclic compounds, they are an important motif of many compounds with medicinal applications [[3], [4], [5], [6]]. The five membered ring triazoles are of special interest due to the diversity of their biological activities as well as the diversity of their synthesis [[7], [8], [9], [10], [11], [12], [13], [14], [15], [16]]. Classes of triazoles such as 1,2,3 and 1,2,4 triazoles are continuously proven to inspire the scientific community [3,11,[16], [17], [18], [19], [20]]. Furthermore, sulfur-linked 1,2,4-triazoles considered an important class of sulfur containing compounds with diverse potential applications in drug discovery, especially 1,2,4-triazole-3-thione ring system [21]. several biologically active 1,2,4-triazole-3-thione derivatives have been reported with broad spectrum of bioactivities such as antioxidant [22], antiviral [23], and anticancer [24]. On the other hand, 1,2,3-triazoles are considered a privileged structure scaffold. This is due to its linker properties and ease of synthesis, diverse 1,2,3-triazole derivatives were prepared and screened for biological activities [25]. Based on the above and in a continuation of our research in the copper(I)-catalyzed alkyne-azide cycloaddition (CuAAC) reaction (click chemistry) [26], we are reporting the synthesis, crystal structure, Hirshfeld surface analysis, and DFT Calculation of 4-(5-(((1-(3,4,5-trimethoxyphenyl)-1*H*-1,2,3-triazol-4-yl)methyl)thio)-4-phenyl-4*H*-1,2,4-triazol-3-yl)pyridine.

## 2 Experimental section

### 2.1 General

All chemicals and reagents were purchased from Sigma-Aldrich and Acros and used without further purification. Melting point was measured with a Stuart melting point apparatus and was uncorrected. Nuclear magnetic resonance (NMR) spectra were measured on a Bruker Avance ІІІ-500 MHz spectrometer, ^13^C spectra were measured at 125 MHz in deuterated dimethylsulphoxide (DMSO-*d*_6_) as a solvent,. Chemical shift values (*δ*) were reported in parts per million (ppm) with reference to residual resonance of the solvent used. High-resolution mass spectrum (HRMS) is measured (in positive) using the electrospray ion trap (ESI pos low mass) technique by collision-induced dissociation on a Bruker APEX-IV (7 T) instrument. Single-crystal X-ray diffraction data were collected using Bruker-D8 Venture single crystal XRD, equipped with (Mo & Cu), X-ray Source (λ = 0.71073 Å) at 100–293 K.

#### 2.1.1 Procedure for the synthesis of 4-(4-phenyl-5-(prop-2-yn-1-ylthio)-4*H*-1,2,4-triazol-3-yl)pyridine (1)

Compound **1** was synthesized according to the published [4,27], in brief: Isoniazid (1equiv.) and phenylisothiocyanate (1.5 equiv.) were refluxed in CH_3_OH for 3hr, where upon completion the solution was cooled, and the precipitate was collected. The produced intermediate (thiosemicarbazide derivative) was refluxed in 2N NaOH. Thereafter, the solution was cooled and acidified with hydrochloric acid to pH = 5–6. Precipitate formed was collected by filtration. The collected precipitate (1equiv.) was mixed with triethylamine (1equiv.) in methanol and stirred at 0–5 °C and propargyl bromide (1equiv.) was added slowly. The reaction mixture was stirred at room and monitored by tlc. Upon completion, the solution was evaporated under vacuum and the precipitate formed was collected and recrystallized from ethanol to afford compound **1**.

#### 2.1.2 Procedure for the preparation of 5-azido-1,2,3-trimethoxybenzene (2)

The desired azide derivative was prepared following the standard published procedure [26], where 1,2,3-trimethoxyaniline (10.0 mmol) was dissolved in cooled HCl_(aq)_ (6.0 mL, 6 M) T = 0–5 °C. To this cold solution, cold solution of sodium nitrite (10.0 mmol) was added, followed by the dropwise addition of an aqueous solution of NaN_3_ (10.0 mmol). The mixture was stirred for 20 min at r.t. The produced azide was extracted with ethylacetate. The organic phase was dried over anhydrous sodium sulfate, filtered and evaporated at room temperature to afford the crude azide which was used without further purification.

#### 2.1.3 Procedure for the preparation of 4-(5-(((1-(3,4,5-trimethoxyphenyl)-1*H*-1,2,3-triazol-4-yl)methyl)thio)-4-phenyl-4*H*-1,2,4-triazol-3-yl)pyridine (3)

The target compound **3**, was synthesized following the published procedure [4], in which, a mixture of **1** (1.0 eq) and azide **2** (272 mg, 1.3 mmol, 1.3 eq) was stirred in DMF (10.0 mL). To this mixture, sodium ascorbate (0.5 mmol) and CuSO_4_·5H_2_O (0.16 mmol) was added. The reaction progress was monitored using tlc, upon completion, the formed precipitate was filtered off and recrystallized from EtOH/H_2_O to produce a nice pure crystalline white product **3**.

Yield 83 %, m. p. 151–152 °C. ^1^H NMR (500 MHz, DMSO-*d*_*6*_): *δ* = 8.77 (s, 1H, triazole H), 8.76–8.50 (br s, 2H, Ar-H), 7.65–7.53 (m, 3H, Ar-H), 7.50–7.73 (m, 2H, Ar-H), 7.30 (br s, 2H, Ar-H), 7.17 (s, 2H, Ar-H), 4.49 (s, 2H, SCH_2_), 3.86 (s, 6H, ArOCH_3_), 3.70 (s, 3H, ArOCH_3_). ^13^C NMR (125 MHz, DMSO-*d*_*6*_): *δ* = 153.98, 152.95, 150.61, 143.85, 137.86, 134.24, 133.80, 132.88, 131.00, 130.65, 128.04, 122.83, 98.60, 60.68, 56.78, 27.29. HRMS (ESI) (*m*/*z*): 502.1661 [M+H]^+^ calcd. C_25_H_24_N_7_O_3_S, found 502.1649.

### 2.2 Results and discussion

**Scheme 1:**
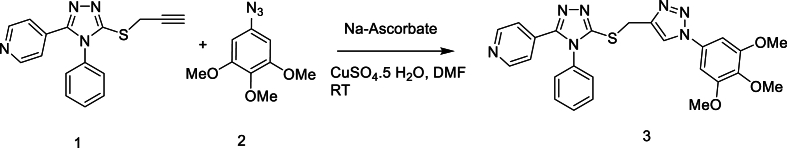
Synthesis of 4-(5-(((1-(3,4,5-trimethoxyphenyl)-1*H*-1,2,3-triazol-4-yl)methyl)thio)-4-phenyl-4*H*-1,2,4-triazol-3-yl)pyridine **3**.

The designed compound was synthesized from the propargyl tagged 1,2,4-triazole derivative **1**^**27**^ through the well-established CuAAC reaction click methodology with the azide derivative **2**^**26**^ as shown in Scheme 1. Compound **3** was isolated as a white solid in 83 % yield. The success of this synthesis was clear from the ^1^H and ^13^C NMR analysis of the produced product, the non-existence of any terminal alkyne proton which is characteristic signal for compound **1** along with the downfield singlet signal resonating at *δ* = 8.77 ppm which is assigned to the H-4 proton of the newly formed 1,2,3 triazole ring, indicated the successful approach and the formation of the new ring system, on the top of that the ^13^C NMR of compound **3** showed no signals for any sp-hybridized carbons. Furthermore, ^1^H NMR of compound **3** showed the three methoxy groups signals resonating at *δ* = 3.86 ppm (6H) and *δ* = 3.70 ppm (3H).

Compound 3 showed moderate activity against *Escherichia coli* ATCC 25,922, and Candida albicans NRRL Y–477 at a concentration of 2 mg/100 μL. No significant cytotoxic activity was observed for compound 3 when tested *in vitro* against human colon carcinoma (HCT116), human cervix carcinoma (HeLa) and human breast adenocarcinoma (MCF7)) at 10 μM concentration [4].

### 2.3 Structure determination

**Fig. 1:**
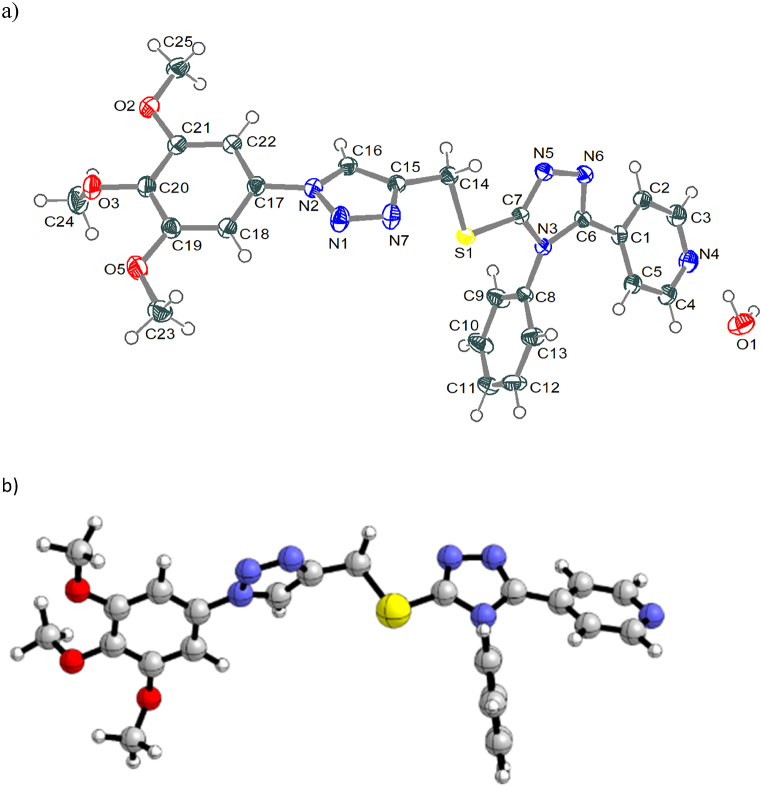
a. The molecular structure of compound **3**. Displacement ellipsoids are drawn at the 30 % probability level. b. Optimized structure at B3LYP level using 6-311+G (2d, p) basis set.

The molecular structure for compound **3** is shown in Fig. 1 with atom labelling. Single crystal of C_25_H_25_N_7_O_4_S was obtained through crystallization of pure compound from ethanol*/water*. A suitable needle-like colorless specimen was selected and mount on Bruker D8-VENTURE diffractometer. The frames were integrated with the Bruker SAINT software package using a narrow-frame algorithm. The crystal was kept at 100(2) K during data collection. Using APEX 5 software, the structure was solved and refined with Bruker SHELXTL Software Package. Further refinements were performed using Olex2. Data were corrected for absorption effects using the Multi-Scan method (SADABS). Structure solution program using Intrinsic Phasing and refined with the SHELXL refinement package using Least Squares minimization. ORTEP plots were generated using ORTEP-3 program (version 2020.1 for windows). The crystal structure data, parameters and measurement conditions corresponding to compound **3** are listed in Table 1.

**Table 1:** Crystal data and structure refinement for compound **3**.

Identification code	CCDC 2355975
Empirical formula	C_25_H_25_N_7_O_4_S
Formula weight	519.58
Temperature/K	100(2) K
Radiation	MoKα (λ = 0.71073)
Crystal system	monoclinic
color	colorless
Crystal description	needle
Space group	*P*2_1_/*c*
a/Å	17.478(3)
b/Å	5.4216(8)
c/Å	25.899(4)
β/°	90.725(5)
Volume/Å^3^	2453.9(6)
Z	4
ρ_calc_ g/cm^3^	1.406
μ/mm^−1^	0.180
F(000)	1088.0
Crystal size/mm^3^	0.376 × 0.085 × 0.055
2Θ range for data collection/°	3.892 to 66.3
Index ranges	−26 ≤ h ≤ 26, −8 ≤ k ≤ 7, −39 ≤ l ≤ 38
Reflections collected	56,601
Independent reflections	9351 [R_int_ = 0.0855, R_sigma_ = 0.0582]
Coverage of independent reflections/%	99.8
Max. and min. transmission	0.9900 and 0.9360
Refinement method	Full-matrix least-squares on F^2^
Data/restraints/parameters	9351/0/346
Goodness-of-fit on F^2^	1.088
Final R indexes [I ≥ 2σ (I)]	R_1_ = 0.0587, w*R*_2_ = 0.1301
Final R indexes [all data]	R_1_ = 0.1089, w*R*_2_ = 0.1714
Largest diff. peak/hole/e Å^−3^	0.38/-0.46

Crystallographic data for the structure of compound **3**, in this study have been deposited with the Cambridge Crystallographic Data Centre under the depository No. CCDC 2355975. Copies of these data can be obtained, free of charge, on application to CCDC, 12 Union Road, Cambridge CB2 IEZ, UK, (fax: +44-1223-336033 or e-mail: deposit@ccdc.com.ac.uk or http://www.ccdc.ac.Uk).

The asymmetric unit of compound **3** comprises a single molecule with one lattice water as depicted in Fig. 1. It is composed of five unsaturated rings, two triazole, one pyridyl and two phenyl rings. The molecule is not planar due to steric repulsion between rings. In the 1,2,4-triazole, the pyridyl group shows angle of 28.35(7)° between the two rings normal plane while the angle is 77.65(8)° between normal plane of the phenyl group attached to C6 and the normal plane of the 1,2,4-triazole. On the other hand, in 1,2,3-triazole the angle is 23.79(7)° between normal plane of the phenyl group attached to N2 and the normal plane of the 1,2,3-triazole. The N–N [N5–N6: 1.390 Å, N1–N7: 1.310 Å; N1–N2: 1.356 Å] and C–N [N2–C16: 1.354 Å, N7–C15: 1.362 Å, C7–N5: 1.315 Å, C7–N3 1.370 Å, C6–N3: 1.378 Å, C6–N6: 1.315 Å] bond lengths of triazole rings are within the values reported for N-N and C-N bonds in triazole rings. The angle around sulfur atom (C14–S1–C7) shows a normal bent angle of 99.20°.

The water molecule of crystallization in **3** form hydrogen bonds with the pyridyl nitrogen N4 with distance of 1.94 Å.

**Fig. 2:**
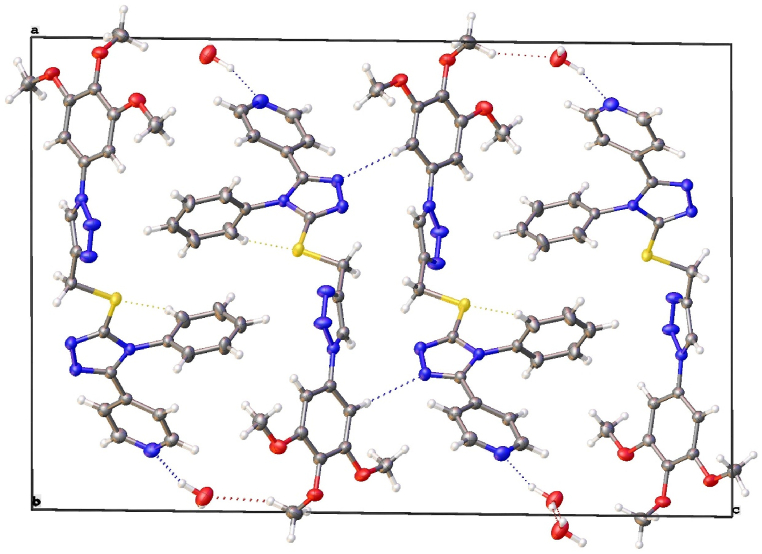
Layers of dimers parallel to ac plane. Hydrogen bonding are shown as dashed lines.

The structure shows more than one hydrogen bonding interactions as shown in Fig. 2. The molecules are linked in the crystal *via* hydrogen bonding interactions between N5 and N6 in 1,2,4-triazole and the hydrogen at 1,2,3 triazole H16(1-X, 1-Y, 1-Z) and a hydrogen on the phenyl at the 1,2,3 triazole H22(1-X, 1-Y, 1-Z) with 2.735 and 2.559(2) Å distance, respectively. Another hydrogen bonding interaction link these dimers between the sulfur atom S1 and hydrogen at phenyl ring attached to 1,2,4-triazole H9 (1-X, 2-Y, 1-Z) with 2.7694(6) Å distance.

### 2.4 Computational studies

Density Functional Theory (DFT) and time dependent Density Functional Theory methods were used to provide an insight into the ground and excited of **3** in gas phase. The structure of the final product **3** was optimized at the level of theory B3LYP using 6-311+G (2d, p) basis set [[28], [29], [30]]. The single point energy of the optimized energy calculations of the gas phases excited state was determined at the same level of theory using the time dependent DFT methods. All DFT calculations were conducted using G16 (Linux) through input files prepared by GV6 software. The obtained X-ray crystal structure was used an input on CrsytalExplorer platform [31] this to determine the Hirshfeld surface analysis, hence providing an insight into the types of interactions between different groups.

#### 2.4.1 Results and discussion

**Fig. 3:**
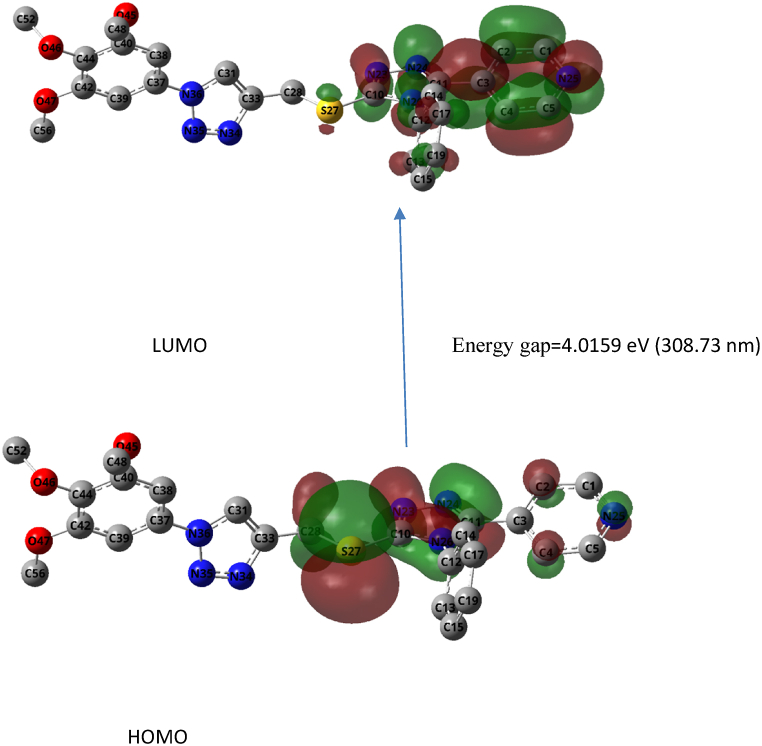
The Frontier Molecular Orbital of the HOMO-LUMO transition where hydrogen atoms are not displayed. Blue: Nitrogen; cray: carbon; Red: Oxygen; Yello:Sulfur.

A comparative structural analysis between the optimized and X-ray structure revealed that there are not any major discrepancies with bond lengths, angles and dihedral angles as shown in Table 2. In addition, the Frontier Molecular Orbital (FMO) analysis using the time dependent density functional theory showed that the major transition occurs from the HOMO-131 (n) to LUMO-132 (π∗) with absorbance of 308.73 nm and oscillator strength of 0.3455, Fig. 3. The transitions from lower HOMO-m to higher LUMO + m are not observed through the TDDFT where the recorded oscillator strengths are zeros.

**Table 2:** Selected parameters of the optimized and X-ray structure of the obtained product.

Parameter	Optimized	X-ray structure (R2)
*Bond lengths (A*^*o*^*)*		
C37-C36	1.42259	1.433(2)
C33-C28	1.48766	1.490(3)
C28-S27	1.8559	1.814(2)
S27-C10	1.75615	1.731(2)
C11-C3	1.4611	1.442(2)
N26-C12	1.43276	1.463(3)
*Angles (Degree)*		
C37-N36-C31	128.9915	128.82(16)
C33-C28-S27	109.17722	106.85(12)
S27-C10-N26	122.51989	120.60(13)
C12-N26-C11	129.89293	130.64(15)
N26-C11-C3	127.21962	126.77(17)
*Dihedral Angles (Degrees)*		
C39-C37-N36-C31	152.17880	148.46(18)
N34-C33-C28-S27	−91.30636	−90.00(15)

**Fig. 4:**
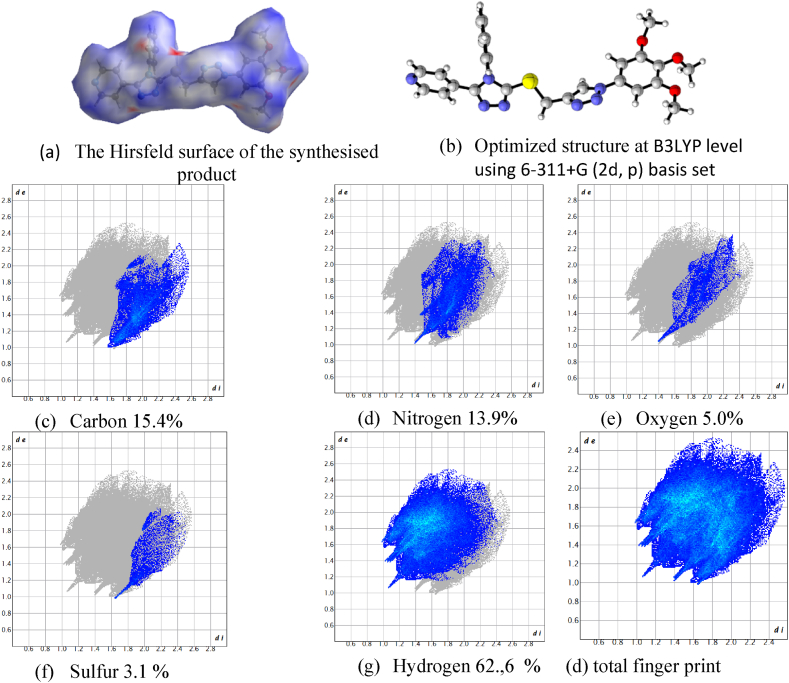
The Hirshfeld surface map of the final product along with comparative of the full and decomposed fingerprints plots of the product along with the optimized structure.

Furthermore, the FMO analysis revealed that the main contribution of the n-type orbital (donor) are the lone pair of electrons on the sulfur atoms. On the other hand, the main constituent of the π∗ (acceptor) are the extended pi system on the aromatic rings. The Hirshfeld analysis showed that the bulk of interaction is due to hydrogen bonding, as the hydrogen atoms constitute the major surface area exposure of 63 %. The sulfur and triazoles nitrogen are exposed to external interactions as revealed in Hirshfeld surfaces as highlighted red signifies the exposure through de and di (the distances to the internal and external distance from surface to the nearest inner and external nucleus), Fig. 4.

**Fig. 5:**
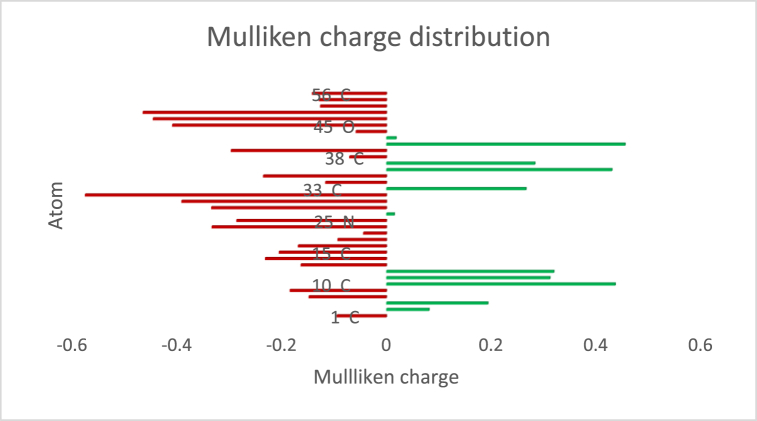
Mulliken charge distribution of the obtained product where the red color represents the negatively charged atoms and the green color represents the positively charged ones. Hydrogen atoms are not listed. The calculated structural and electronic determinations are in full agreement with experimental results of the obtained product.

The Mulliken charges distribution showed that the negative charges are located on sulfur and triazole nitrogens, Fig. 5. The N36 possessed a positively Mulliken positive charge due to the electron withdrawing effect of negatively charged oxygen on the adjacent phenyl groups. In addition, all hydrogen atoms were positively charged.

## 3 Conclusion

The electronic and molecular properties of the optimized product **3** through the DFT and TDDFT calculation along with Hirshfeld surface analysis of X-ray structure, could provide a clear insight about the role of sulfur and triazole nitrogen in the crystal packing and reactivities. The C-H ….N interactions was confirmed by the analysis of the fingerprint plots of the Hirshfeld molecular surface where that H-H contact contribution was recorded to be 62.6 %. Moreover, Hirshfeld surface analysis also showed the reactive electrophilic and nucleophilic sites are located around the nitrogen and hydrogen atoms respectively. In addition, the optimized molecular structure using DFT methods with high level of theory and basis set was in great agreement with X-ray determined structure which provided an additional affirmation of the reached conclusions and results. The FMO analysis predicted that the HOMO- LUMO gap to be 4.0159 eV which indicated the great extent of stability of the obtained structure.

## CRediT authorship contribution statement

**Mohamed El-Naggar:** Writing – review & editing, Writing – original draft, Methodology, Funding acquisition, Conceptualization. **Kamrul Hasan:** Writing – review & editing. **Monther A. Khanfar:** Writing – review & editing, Software, Methodology, Investigation, Conceptualization. **Fatima-Azzahra Delmani:** Writing – review & editing, Data curation. **Ihsan A. Shehadi:** Writing – review & editing, Writing – original draft, Validation, Software, Methodology. **Raed Al-Qawasmeh:** Writing – original draft, Methodology, Investigation, Conceptualization. **Hussein M. Elmehdi:** Writing – review & editing, Methodology, Data curation.

## Declaration of competing interest

The authors declare that they have no known competing financial interests or personal relationships that could have appeared to influence the work reported in this paper.
